# Spatiotemporal variation in mechanisms driving regional‐scale population dynamics of a Threatened grassland bird

**DOI:** 10.1002/ece3.3004

**Published:** 2017-04-27

**Authors:** Danielle M. Ethier, Nicola Koper, Thomas D. Nudds

**Affiliations:** ^1^ Department of Integrative BiologySummerlee Science ComplexUniversity of GuelphGuelphONCanada; ^2^ Natural Resources InstituteUniversity of ManitobaWinnipegMBCanada

**Keywords:** agricultural ecosystems, Bobolink (*Dolichonyx oryzivorous*), population trends, spatial, species‐at‐risk, temporal

## Abstract

To achieve national population targets for migratory birds, landscape‐level conservation approaches are increasingly encouraged. However, knowledge of the mechanisms that drive spatiotemporal patterns in population dynamics are needed to inform scale‐variant policy development. Using hierarchical Bayesian models and variable selection, we determined by which mechanism(s), and to what extent, changes in quantity and quality of surrogate grassland habitats contributed to regional variation in population trends of an obligatory grassland bird, Bobolink (*Dolichonyx oryzivorous*). We used North American Breeding Bird Survey data to develop spatially explicit models of regional population trends over 25 years across 35 agricultural census divisions in Ontario, Canada. We measured the strength of evidence for effects of land‐use change on population trends over the entire study period and over five subperiods. Over the entire study period, one region (Perth) displayed strong evidence of population decline (95% CI is entirely below 0); four regions displayed strong evidence of population increase (Bruce, Simcoe, Peterborough, and Northumberland). Population trends shifted spatially among subperiods, with more extreme declines later in time (1986–1990: 28% of 35 census divisions, 1991–1995: 46%, 1996–2000: 40%, 2001–2005: 66%, 2006–2010: 82%). Important predictors of spatial patterns in Bobolink population trends over the entire study period were human development and fragmentation. However, factors inferred to drive patterns in population trends were not consistent over space and time. This result underscores that effective threat identification (both spatially and temporally) and implementation of flexible, regionally tailored policies will be critical to realize efficient conservation of Bobolink and similar at‐risk species.

## 1 Introduction

Landscape‐level approaches are recognized as important to migratory bird conservation (Martin & Finch, 1995). In practice, however, large‐scale conservation plans are rarely implemented, perhaps due to a lack of knowledge about the mechanisms that drive patterns in species population dynamics at intermediate scales amenable to policy development and implementation (e.g., counties, management units, municipalities). Regional approaches linking avian population dynamics to environmental covariates are therefore important to identify causes of avian population fluctuations and inform development of scale‐variant policies.

Birds dependent on agricultural habitats for breeding have exhibited more significant population declines than birds of any other habitat type in North America (Herkert, 1995; Knopf, 1994; Peterjohn & Sauer, 1999). Considerable geographic variation exists in these trends (Sauer, Hines, & Fallon, 2005), and its drivers remain largely unresolved (Corace, Flaspohler, & Shartell, 2009). Given that the majority of native grassland ecosystems have been converted to agriculture (Fletcher & Koford, 2002), regional variation in agricultural practices is plausible explanations for geographic variation in avian population dynamics (Hill, Egan, Stauffer, & Diefenbach, 2014).

Correlations among changing farming practices and avian population trends are often used to identify potential drivers of population dynamics and inform the growing interest in landscape‐level conservation approaches (e.g., North American Waterfowl Management Plan, Partners in Flight, The Nature Conservancy's Migratory Bird Program). Such relationships are poorly understood for many species (Chamberlain, Fuller, Bunce, Duckworth, & Shrubb, 2000), and/or best resolved at broad geographic extents (i.e., nation, state/province, or Bird Conservation Region). For example, research into the causes of state‐scale patterns in grassland bird population declines implicates changes in habitat amount (Hill et al., 2014; Murphy, 2003; Perlut, 2014), pesticide use (Mineau & Whiteside, 2013), factors correlated with increasing human populations, and declines in the number of dairy farms (Perlut, 2014). While these results suggest large‐scale shifts in population trajectories and underlying environmental processes, they may not reflect patterns and processes at smaller scales (Herkert, 1995). Specifically, results from broad‐scale analyses could lead to incorrect inferences about threats to population persistence if (1) aggregating data at broad scales homogenizes regional variability in trend estimates (Bled, Sauer, Pardieck, Doherty, & Royle, 2013), or (2) the factors that are influential vary over time (Morris, 1994). Modeling frameworks are therefore needed that take into account spatiotemporal variability in landscape pattern and process to provide robust insights into the mechanisms driving avian population dynamics, and better inform policy development to achieve landscape‐scale conservation targets (Flather & Sauer, 1996).

**Figure 1:**
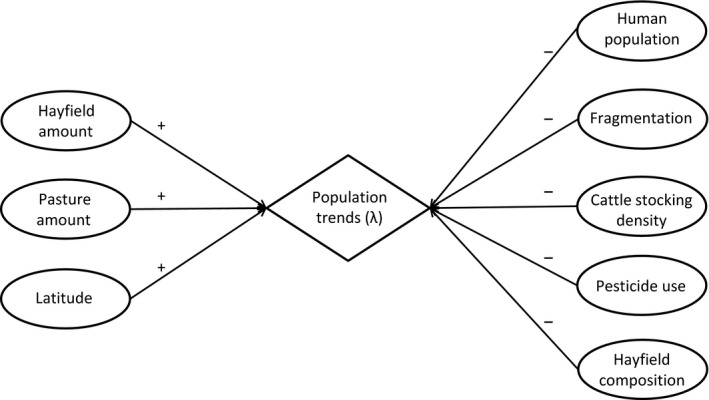
Directed acyclic graph showing causal relationships (link and arrows) among variables (ovals) hypothesized to account for population declines in Bobolink (*Dolichonyx oryzivorous*) (diamond) based on evidence in the published literature and/or stakeholder consultation (detailed in Appendix S1). Positive (left) and negative (right) indicators specify the predicted direction of the cause–effect relationship

Bobolinks (*Dolichonyx oryzivorous*) are among many species of grassland birds that have declined over much of their breeding range in the past several decades, resulting in their listing as a species of conservation concern in both Canada and the United States (COSEWIC, 2010; U.S. Fish and Wildlife Service, 2008). Like other grassland birds, Bobolinks are now dependent on surrogate grassland habitats in agro‐ecosystems for breeding (Hunter, Buehler, Canterbury, Confer, & Hamel, 2001), which implicates changes to land‐use practices during this season as potential drivers of population change. Murphy (2003) identified positive correlations between Bobolink population trends and hayfield amount and negative correlations with pasture area. At the regional‐scale, Bobolink population trends vary spatially and temporally (Ethier & Nudds, 2015), suggesting that variation in land management practices at this scale, rather than more broadly, may contribute to population change. We therefore tested predictions under eight alternative hypotheses about factors driving regional variation in Bobolink population trends, to determine whether, where, and to what spatiotemporal extent changes in the quantity or quality of surrogate grassland habitats contributed to population declines. Specifically, we assessed how changes in habitat amount (hayfields and pastures, separately), hayfield composition, cattle stocking density, pesticide use, human population growth, habitat fragmentation, and factors that covary with latitude drive spatiotemporal fluctuations in Bobolink population trends (Figure 1; detailed hypotheses and predictions in Appendix S1).

## 2 Methods

### 2.1 Study area

The study area covered 97,136 km^2^ of the Ontario portion of North American Bird Conservation Region 13 (43°69′N 79°45′W) within the mixedwoods plains ecozone (Crins, Gray, Uhlig, & Wester, 2009). The majority of this region has been converted to agriculture (Baldwin, Desloges, & Band, 2013) and urban uses (Crins et al., 2009). The dominant agricultural sectors include fruit, row and forage crops, poultry, hogs, and beef and dairy cattle (Government of Ontario, 2013). An estimated 1.5 million hectares are surrogate agricultural grasslands (hayfield and pasture), the management of which is highly influenced by economic market forces (Agriculture and Agri‐Food Canada, 2011). These surrogate grasslands support the greatest abundance of breeding Bobolink nationally, at 10%–12% of the global breeding population (COSSARO, 2010). Bobolink are listed as a threatened species within Ontario as they have exhibited more precipitous declines here than in other provinces in Canada or in the USA (ESA, 2007). As a result, there is significant interest in understanding the factors driving regional variation in abundance to tailor effective and efficient conservation practices across the province.

### 2.2 Response variable

To facilitate the use of Agricultural Census data as predictor variables, we adapted our previous analysis of Bobolink population trends (Ethier & Nudds, 2015) to align spatially and temporally with available agricultural land‐use data (Statistics Canada 2011). Specifically, we summarized Bobolink trends to the extent of the agricultural census division boundaries (area range: 964–7441 km^2^) over 25 years (1986–2011) and over five subperiods corresponding to census periods: 1986–1990, 1991–1995, 1996–2000, 2001–2005, and 2006–2011. Data from the North American Breeding Bird Survey (BBS), available from the Canadian BBS Results webpage (Environment Canada 2010), were used to generate trends. The BBS comprises a series of 39.4‐km roadside surveys performed once annually by volunteers, who identify and count all birds seen or heard during a 3‐min period, at 50 stops approximately 0.8 km apart (Robbins, Bystrak, & Geissler, 1986). The annual sum of Bobolinks counted over the 50 stops were used as an index of counts.

We used hierarchical Bayesian models with small‐area estimation to generate spatially explicit estimates of population trends. The primary advantage of small‐area estimation is that it permits more precise trend estimates for areas with limited or no data. The adjacency of neighboring areas facilitates borrowing information from one area, effectively increasing the sample size on which trend estimates are based in another (Bled et al., 2013), helping to improve the power of the model to predict trends by accounting for underlying geographic variation in the data (Bahn, O'Connor, & Krohn, 2006).

Each route was assigned to the census division in which the start point for the route was located. Counts of Bobolink ϒ_*i,t*_ were modeled on route *i* in year *t* by a Poisson distribution with a mean λ_*i,t*,_ which depends on the year‐specific intercept (α_*t*_), an observer effect (ω_*K(i,t)*_) for each individual (*K*) carrying out the survey, and a spatial effect (*b*_*c(i),t*_) at the level of the census division (*c*):

$$\gamma_{i,t}∼\text{Poisson}\left(\gamma_{i,t}\right)\text{with}\;\log\left(\gamma_{i,t}\right)=\alpha_{t}+b_{c(i),t}+\omega_{K(i,t)}$$

A customary vague prior was used for the year effect parameters:

$$\alpha_{t}∼\text{Normal}\left(0,0.01\right)$$

Observer effects were assumed to be normal distributed random variables with a mean 0 and variance σ^2^_Obs_:

$$\omega_{K(i,t)}∼\text{Normal}\left(0,\sigma_{\mathrm{Obs}}^{2}\right)\text{with}\sigma ∼\text{Unif}\left(0,10\right)$$

Spatial effects *b*_*c(i),t*_ were modeled by applying a Gaussian conditional autoregressive (CAR) model (Besag, York, & Mollié, 1991) and were allowed to vary for each subperiod separately. Neighborhood weights in a CAR model, where the spatial process is modeled over irregular regions, are generally informed deterministically using adjacency‐based methods (White & Ghosh, 2009) and are widely applied in small‐area estimations for landscape‐scale analyses in the fields of agriculture, epidemiology, ecology, and census surveys (e.g., Bled et al., 2013; He & Sun, 2000; Lawson, Browne, & Rodeiro, 2003; Roa, 2003; Thogmartin, Sauer, & Kuntson, 2004). We assigned 1 to areas that shared a common boundary, and 0 otherwise. This neighborhood structure assumes that the spatial structure random effect for a given region has a mean equal to the average of the random effects of bordering areas and does not depend on distance between areas. Population trends (Δ_*c,a,b*_) could then be defined as the yearly change from year *t*_*a*_ to year *t*_*b*_ for census division *c* expressed as a percent:

$$\Delta_{c,a,b}=100\cdot \left[{\left(n_{c,tb}/n_{c,ta}\right)}^{(1/\left(t_{b}-t_{a}\right))-1}\right]$$

where *n*_ct_ would represent the counts at time *t* of a theoretical route in cell *c* and is specified as:

$$n_{ct}=\exp\left(\alpha_{t}+b_{c,t}+1/2\sigma_{\mathrm{obs}}^{2}\right)$$

The observer variance component (0.5 × σ^2^_Obs_) is the back transformed mean of the observer random effect (Smith, Hudson, Downes, & Francis, 2014).

**Figure 2:**
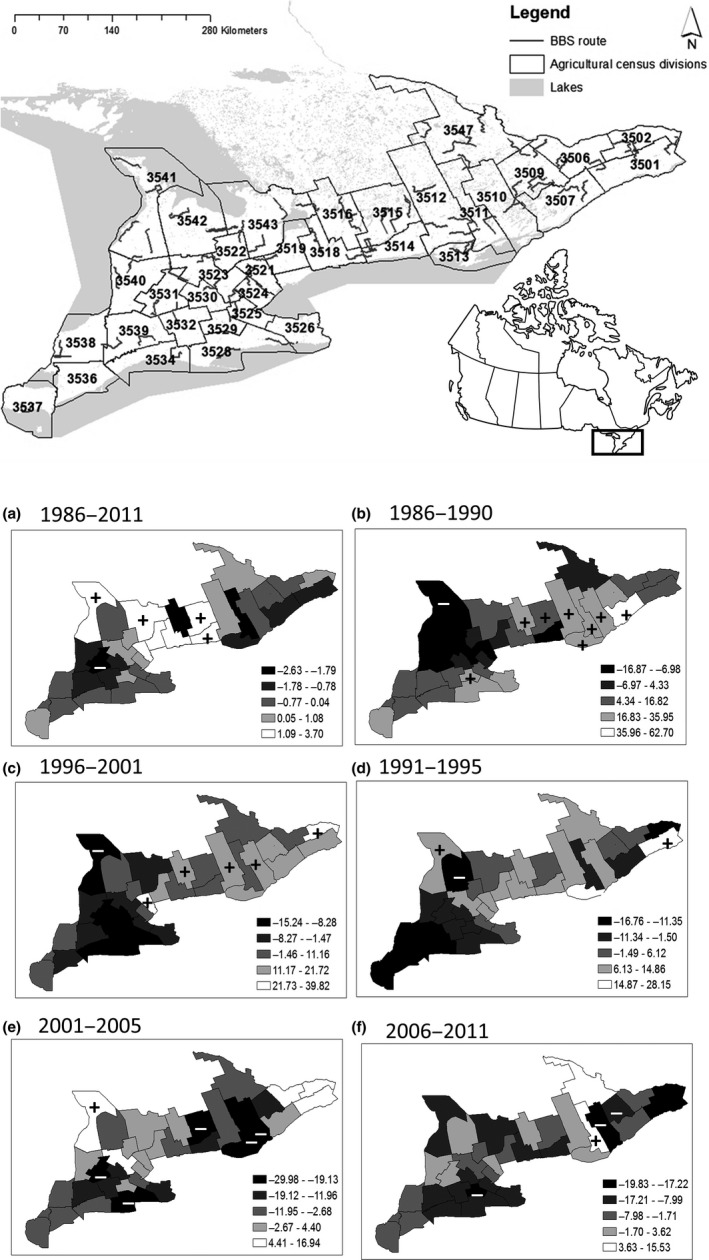
Distribution map of North American Breeding Bird survey routes (BBS) across 35 agricultural census divisions in Ontario, Canada (insert top). Posterior mean population trend estimates for Bobolink (*Dolichonyx oryzivorous*) over the entire study period (a) and by subperiod (b–f) are mapped to agricultural census divisions using spatially explicit hierarchical models in a Bayesian statistical framework. The indicators specify whether the 95% credible interval was above (**+)** or below (**−)** the zero mean population trend line

The model was fitted by iteration using WinBUGS 1.4.3 (Lunn, Thomas, Best, & Spiegelhalter, 2000). Three chains were run to draw 50,000 samples, discarding the first 10,000 iterations. Samples were thinned by 1 in 3 to reduce autocorrelation and obtain regional mean trends (Δ_*c,a,b*_) from the remaining posterior distributions. Convergence was checked using the Gelman–Rubin diagnostic for assessing convergence (maximum R‐hat = 1.2; Brooks & Gelman, 1998) and WinBUGS traceplots. Significant posterior mean trends were interpreted as those with 95% credible intervals (CRI) that did not include zero (Bled et al., 2013). In total, 64 survey routes distributed among 35 agricultural census divisions were used to estimate Bobolink population trends (Figure 2, insert).

### 2.3 Predictor variables

Direct measures (i.e., raw values) were used to reflect mechanistic hypotheses of Bobolink population change when land‐use statistics were available (e.g., change in habitat amount). In instances when direct measures of mechanism were not available (e.g., cattle stocking density, hayfield composition), or hypotheses reflected aggregated mechanisms (i.e., human population growth), we derived indices which best approximated mechanistic hypotheses (Appendix S1).

Hayfield and pastureland amount, cattle density, and hayfield composition within each census division and within 5‐year sampling periods were derived from the Census of Agriculture (Statistics Canada, 2011). Hayfield area was calculated directly by combining alfalfa, alfalfa mix, and tame hay lands. Pasture area was also calculated directly by combining improved and unimproved pasture lands. As cattle stocking density (i.e., number of cattle scaled to the area of pasture lands) and hayfield composition (i.e., the amount of alfalfa hay scaled to area of hay lands) are not directly measured by the agricultural census, we derived indices to approximate these predictors. Specifically, we used ordinary least squares to regress the number of cattle (dairy and beef, combined) on the area of pasture land for each agricultural census division. Model residuals were used as an index of cattle stocking density, as regression‐based approaches to deriving indicators is preferred over the use of ratios (see Allison, Paultre, Gordan, Poehlman, & Heymsfield, 1995). Our index of cattle stocking density therefore reflects the hypothesis that increasing the number of cattle grazed per area of pasture can directly and indirectly affect Bobolink trends by increasing incidents of nest disturbance (e.g., MacDonald, 2014) or degrading nesting habitat (e.g., Owens & Myres, 1973), respectively (detailed in Appendix S1). An index of hayfield composition was calculated in a similar fashion, as the residual variation in area of alfalfa hay regressed on the total area of all hay types. These residuals reflect the hypothesis that increasing amounts of alfalfa‐dominated hay relative to other hay types can have two important effects on Bobolink (detailed in Appendix S1). In short, alfalfa‐dominated fields are cut earlier in the season, putting Bobolink at greater risk of nest loss (Barnes, Nelson, Moore, & Collins, 2007), and alfalfa‐dominated fields are less attractive to Bobolinks, effectively lessening the amount of available habitat (habitat loss; Bollinger, 1995).

A static measure of habitat fragmentation was derived using data from Southern Ontario Land Resource Information System (SOLRIS; Ontario Ministry of Natural Resources, 2008), a raster‐based inventory of 23 land cover types at 15‐m^2^ resolution, developed with satellite imagery collected circa 2001. A static fragmentation index was used since temporally explicit geospatial land‐use data at the extent of our study area are not available to assess fragmentation as a dynamic process. We reclassified the 23 land‐use categories into a binary classification of habitat/nonhabitat following Smith, Fahrig, and Francis (2011), in which all open vegetated habitat types (natural grassland, pasture, abandoned fields, croplands) were combined into a single class of suitable habitat (Corace et al., 2009). To index the degree of fragmentation, we used the normalized Landscape Shape Index (nLSI) from FRAGSTATS version 4 (McGarigal, Cushman, & Ene, 2012), a standardized measure of edge density adjusted for the size of the landscape. This indicator was selected because it is only weakly correlated with habitat amount, can be discriminated from among landscapes with different spatial aggregations (Wang, Blanchet, & Koper, 2014), and consistently and robustly scales in relationship with landscape grain (Wu, 2004).

From the Survey of Pesticide Use in Ontario, we extracted estimates of active pesticide ingredients for major crop types (i.e., field, fruit, and vegetable crops) (Appendix IX, McGee, Berges, & Beaton, 2010). Every 5 years, quantities of active ingredients are computed by multiplying the area sprayed, times the concentration, times the application rate for different crop types. From this, a reliable provincial level estimate of total active ingredients can be allocated among regions on the basis of area of crop grown (McGee et al., 2010). Using the provincial land area estimates from the Census of Agriculture (Statistics Canada 2011), we derived an index of active ingredients used, per hectare, per major crop type for each census year. We used this value as a multiplication factor to calculate the total amount of pesticides used in each agricultural census division in a given year based on the area of different crop types grown.

Change in human population size (i.e., number of people) was used as an index of human activities that result in bird mortality (e.g., collisions with vehicles, secondary effects of roads on adjacent habitats; see Appendix S1). Human population size in each agricultural census division was retrieved from the Census of Population (Statistics Canada 2014), which is conducted in the same years as the Agricultural Census. Data were accessed using the Canadian Census Analyser from the University of Toronto's Computing in the Humanities and Social Sciences online data centre (CHASS, 2010).

We calculated change in predictors over the entire study period (1986–2011), and for each subperiod (1986–1990, 1991–1995, 1996–2000, 2001–2005, and 2006–2010), as a percent change from the previous census year as [(year_*x*_ value – year_*x*−1_ value)/|year_*x*−1_ value|) × 100] following Murphy (2003) and Perlut (2014).

### 2.4 Analysis

We used Bayesian variable selection to measure the strength of evidence that environmental covariates (percent change or static measures in the case of latitude and fragmentation) for each subperiod and the whole time period were good predictors of trends in Bobolink abundance (response) (Mitchell & Beauchamp, 1988). Bayesian variable selection was chosen because it is well suited to situations where the sample size is small relative to the number of predictors (O'Hara & Sillanpää, 2009). We fit a full subset linear regression model of the form:

$$yi=\sum_{j=1}^{p}\beta_{j}I_{j}x_{ij}+\varepsilon_{i}$$

where *yi* corresponds to the index of counts, β the regression coefficients of the known *j*‐th environmental covariate, *x*_*ij*_ is the known *j*‐th covariate for the *i*‐th census division, and ε is a random error term. The embedded indicator variable *I*_j_ for the *j‐*th regression coefficient is supported at two points, 1 and 0, and is the tool for variable selection. If *I*_j_ takes the value 1, the *j‐*th regression coefficient is considered a relevant predictor of the response, and the value 0 otherwise. The regression model is fitted with a Bayesian approach, which required explicit statements of prior distribution on all unknown quantities. We assume ε_i_ follows a normal distribution, *N*(0, σ^2^), and independent priors for β, *I,* and σ; where β is assigned a multivariate normal distribution; *I*_*j*_ for *j* = 1,…, *p* are chosen independently, each with a prior Bernoulli distribution of 0.25 (Chipman, Hamada, & Wu, 1997; Meyer & Box, 1992; Meyer & Wilkinson, 1998); and σ has an inverse gamma distribution (Kuo & Mallick, 1998). Priors are updated with the data by means of the Markov Chair Monte Carlo (MCMC) method. When the posterior distribution of *y*_*j*_ *≥* 0.25 (i.e., the activation probability), the *j*th environmental covariate is considered an important predictor of the response (O'Hara & Sillanpää, 2009).

The regression model was also fit with a 0.5 prior on the Bernoulli indictor (Royale & Dorazio, 2008) to determine whether the assignment of this prior affected the selection of likely important predictor variables. Results were robust to the selection of Bernoulli prior (unpublished data). We therefore retained 0.25 as our prior, which reflects the assumption that a majority of predictor variables are likely unimportant to the outcome of the response. This is a common assumption in ecological studies (e.g., Mutshinda, Finkel, & Irwin, 2013), particularly when predictor variables are derived proxies rather than direct environmental measures (Mundry, 2011).

Bayesian variable selection is generally robust to correlated predictors (Mutshinda et al., 2013), although very high collinearity (>0.90) may influence results (Ghosh & Ghattas, 2015). We therefore checked all correlation coefficients prior to analysis. All predictors were centered and scaled using the mean and standard deviation to improve the performance of MCMC and allow for comparisons among predictors (Gilks & Richardson, 1995). Variable selection was implemented in WinBUGS 1.4.3 (Lunn et al., 2000) using BugsXLA graphical user interface (Woodward, 2011). For the entire study period, and each of the temporal subperiods, we ran three chains, thinned by 1 in 3, and based our inference on 50 000 samples from the posterior distribution of parameters, after 5 000 interactions were discarded. Convergence was checked using the Gelman–Rubin diagnostic in WinBUGS (Brooks & Gelman, 1998).

## 3 Results

### 3.1 Spatial–temporal population trends

Mean trends over the entire study period (1986–2011, Figure 2a) were significantly negative in one of 35 census divisions (Perth), and positive in four regions (Bruce, Simcoe, Peterborough, and Northumberland). Population trends shifted spatially among temporal subperiods (Figure 2b‐f). Generally, the number of regions with negative mean trends increased over time (1986–1990: 28%, 1991–1995: 46%, 1996–2000: 40%, 2001–2005: 66%, 2006–2010: 82% of 35 census divisions). Several regions experienced significant population declines in one or more subperiod (Grey, Hamilton, Lanark, Perth), but more experienced increases (Stormont‐Dundas‐Glengarry, Prescott‐Russell, Leed‐Grenville, Hastings, Price Edward, Northumberland, Kawartha Lakes, Peel, Simcoe), particularly in the northeastern portion of the study area. Several regions also oscillated between periods of significant increases and decreases over the entire study period (Frontenac, Lennox‐Addington, Peterborough, Brant, Bruce).

### 3.2 Spatialtemporal landscape change

**Figure 3:**
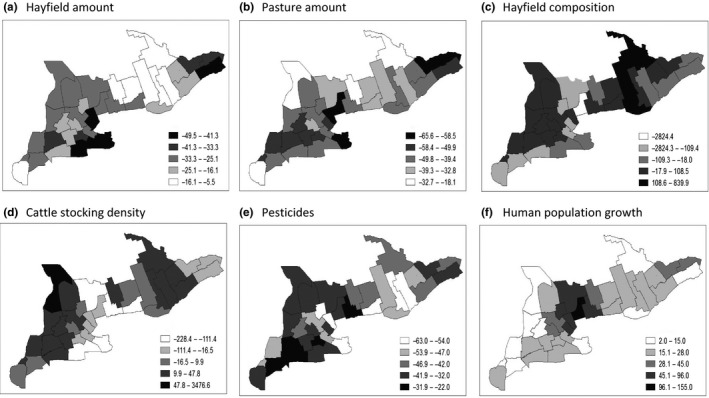
Rates of change in predictor variables over the entire study period (1986–2011) mapped to agricultural census divisions in southern Ontario, Canada

Between 1986 and 2011, there were ubiquitous declines in the amount of hayfield (mean = −25%, range: −50% in Peel to −5% in Hastings; Figure 3a) and pasture lands (mean = −43%, range: −65% in Prescott‐Russell to −18% in Essex; Figure 3b). Habitat fragmentation was least in Essex and greatest in Perth and surrounding regions. The amount of alfalfa hay relative to all hay types increased in 19 of 35 census divisions (Figure 3c); however, the overall ratio declined (mean = −61%, range: −282% in York to 838% in Hastings, one outlier removed). Cattle density increased in 18 of 35 census divisions, of which Bruce had a large influence on the overall positive trend (Figure 3d; mean = 77%, range: −228% in Niagara to 47% in Bruce, one outlier removed). On average, pesticides used on all crop types declined (mean = −42%, range: −63% in Northumberland to −22% in Chatham‐Kent). The slowest rates of decline were clustered in southwestern Ontario (Figure 3e). Human populations grew on average 33% (range: 2% in Chatham‐Kent to 155% in York) and were concentrated in the census divisions adjacent to the Greater Toronto Area (GTA).

Similar to population trends, spatial patterns in landscape composition and land‐use intensification varied temporally. For example, hayfield loss was apparent across all subperiods with the exception of 2001–2005, when they increased briefly (mean = 4%, range: −10% in Peel to 37% in Chatham‐Kent). Pasture loss was also apparent in all subperiods, except from 1991 to 1995 (mean = 5%, range −21% Prescott‐Russell to 98% in Essex). Hayfield composition shifted from increases in alfalfa‐hay from 1986 to 1990 (mean = 17%, range: −214% in Halton to 427% in Hasting) to decreases in later time periods (mean = −117% per year). Similarly, cattle stocking density increased briefly from 1986 to 1990 (mean = 7%, range: −88% in Northumberland to 351% in Bruce), and has been on a steady decline since (mean = −5% per year). Only in Bruce did cattle stocking density experience consistent increases over the course of our study period. Pesticide use shifted from high rates of decline from 1986 to 2001 (mean = −20%, range: −24% in 1996–2000 to −19% in 1986–1990), to increases between 2001 and 2005 (mean = 10%, range −20% in Hastings to 45% in Niagara). Human population growth was greatest from 1986 to 1990 (mean = 8%, range = 4% in 1996–2000 to 12% in 1986–1990) and least during the most recent census periods (mean = −3%, range −13% in York to 4% in Chatham‐Kent).

### 3.3 Bayesian variable selection

**Figure 4:**
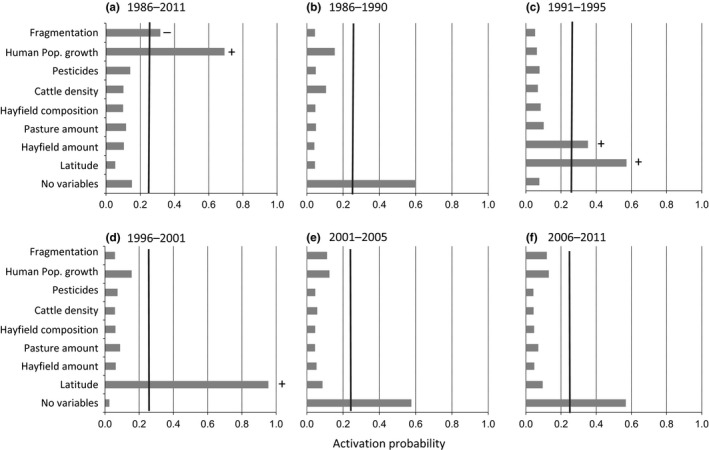
Plots depicting likely important predictors of spatialtemporal patterns in Bobolink (*Dolichonyx oryzivorous*) population trends using Bayesian variable selection. Posterior activation probabilities ≥25% are considered evidence that the predictor influenced the outcome of the response (black vertical line). Positive and negative indicators specify the direction of the regression coefficient associated with these variables. No variables indicate the number of none active variable selections terms

Pearson's correlation coefficient between predictors ranged from −0.71 to 0.51, so all variables were included in the analysis. Important predictors of spatial–temporal patterns in Bobolink population trends can be inferred from the plots of posterior probabilities of variable activity (Figure 4). Over the entire study period (1986–2011), human development was positively correlated with Bobolink population trends, and to a lesser extent, fragmentation was negatively correlated with Bobolink trends (Figure 4a). By subperiod (Figure 4b–f), the likely important predictors shifted from latitude and hayfield amount in 1991–1995, to latitude in 1996–2001; none of the predictors we included were selected as likely important in 1986–1990, 2001–2005, and 2006–2011. Hayfield amount and latitude were both positively correlated with Bobolink population trends in all instances.

## 4 Discussion

Our results demonstrate that analyses performed at broad geographic extents have the potential to homogenize regional variability in population trends, which may diminish our ability to identify regions and factors responsible for avian population declines (Bled et al., 2013; Corace et al., 2009; Ethier & Nudds, 2015). For example, at the geographic extent of the province, Bobolinks declined 3% annually from 1967 to 2008 (COSEWIC, 2010). However, when assessed regionally, Perth was consistently identified as an important region contributing to provincial declines (results herein and Ethier & Nudds, 2015), whereas Bruce, Simcoe, Peterborough, and Northumberland displayed evidence of Bobolink population growth. Analyses performed over long timespans can also homogenize important temporal variability in correlations between response and predictors (Lusk, Guthery, George, Peterson, & DeMaso, 2002; Saether & Bakke, 2000). Notably, apparent drivers of regional population change over the entire study period (human population growth and fragmentation) did not emerge as important factors in any of our temporal subperiods. This may be due to nonlinear change or nonstationarity in factors affecting trends, which are otherwise commonly not taken into account among landscape‐scale trend analyses (Hill et al., 2014; Mineau & Whiteside, 2006; Murphy, 2003; Perlut, 2014). Violations of assumptions of linearity and stationarity could lead to imprecise and/or inaccurate estimates of the risks that various threats pose to a population (Hill et al., 2014; Lusk et al., 2002). By allowing the slope of the relationship between response and predictors to vary by temporal subperiod, these potentially confounding effects can be partially accommodated. Results from temporally divided analyses may therefore provide additional insight into the processes that drive regional patterns in avian population dynamics at finer‐temporal scales, in addition to analyses over broader time spans.

The positive relationship between Bobolink and human population trends over the entire study period is inconsistent with the hypothesis that the multiple processes by which humans could negatively affect Bobolink act at the spatialtemporal scales selected here. Conversely, Perlut (2014) identified human population growth as the most important predictor of Bobolink population declines in six northeastern United States over 41 years (mean state‐scale human population growth rate = 33%, range: 7% New York to 68% New Hampshire). Perlut (2014) found human population growth was negatively correlated with all agricultural variables (−0.92 to −0.99), with the exception of total corn acreage (0.10), and concluded that human population growth was at least partially responsible for the loss of cattle‐based agriculture. The latter was also a good predictor of other grassland bird population declines, with the exception of Bobolink. Our correlations between agricultural statistics and human population growth, in contrast, were low (−0.37 to 0.15), suggesting that the observed positive relationship may result from the maintenance of farmland, natural areas, and less intensive agricultural practices in proximity to urban centers. The Greenbelt Protection Act (Legislative Assembly of Ontario, 2004) may have contributed to this observed trend. The Greenbelt retains 1.8 million acres of green space, farmlands, and wetlands in rural areas surrounding the GTA. Since its establishment, additional cost‐share funds have been provided to farmers in the affected areas to improve water quality, wildlife protection, and reduce pesticide application. Consequently, the Greenbelt differs from areas elsewhere in the province; the proportion of organic farms has increased, smaller farm sizes are maintained, and declines in cattle farming are slower (Di Poce, Goarley, & Mausberg, 2009).

High levels of habitat fragmentation were also linked to long‐term Bobolink population declines, which suggest area sensitivity and edge effects may suppress density and nest success, as it does for other grassland birds (Helzer, 1996; Johnson & Temple, 1990). These effects may accumulate with time, since a Bobolink's decision to return to, and breed at, a site is influenced by its own reproductive success, and that of others, in the previous year (Bollinger & Gavin, 1989). Thus, while our measure of fragmentation is static, the demographic processes it influences (i.e., apparent survival) could be cumulative, creating the negative trends we observe. If a temporally varying measure of landscape fragmentation were available, we would expect this effects to be exacerbated. The long‐term viability of Bobolink may therefore depend on the maintenance of large tracts of surrogate grassland habitat where they currently exist, and/or restoring open habitat types in regions where they have been lost.

When our analysis was temporally segregated over five subperiods, Bobolink population trends varied widely (Figure 2b–f), similar to patterns observed for Cerulean warbler (*Setophaga cerulean*) and Red‐bellied woodpecker (*Melanerpes carolinus*) over similar spatial and temporal scales (Bled et al., 2013). Factors associated with these shifts differed from our full time series analysis and included changes in hayfield area and factors that covaried with latitude.

Between 1991 and 1995, Bobolink population growth rates were positively correlated with the amount of hayfield, which is consistent with other lines of evidence. For example, Murphy (2003) found 92% (*n* = 25) of grassland birds exhibited at least one association between population trends and habitat amount; for Bobolinks, this relationship was with hayfield amount. Given that Bobolinks are upwards of four times more likely to be found in hayfields than any other surrogate grassland habitat type (Bollinger, Bollinger, & Gavin, 1990), the maintenance of hayfields on the landscape, regardless of composition, will likely be important to Bobolink conservation.

Bobolink population growth rates were positively correlated with latitude during the 1990s, consistent with the hypothesis that climate‐driven gradients in plant maturation (hence, forage harvest timing) may contribute to spatial patterns in Bobolink population trends. In an average year, optimal hay harvest timing in Ontario is more than 3 weeks earlier in the southwest than in the northeast (J. J. Nocera, pers. commun.), similar to patterns found in Michigan (Corace et al., 2009). However, Bobolink fledgling dates only vary by 1 week over this same geographic gradient (Nocera pers. commun.). As a result, northern Bobolinks are expected to have higher reproductive output in hayfields compared to their southern counterparts. This finding suggests a one‐size‐fits‐all approach to managing Bobolink across the landscape may not be an optimal strategy. For example, in the southern regions of the province, delaying hay harvest to improve Bobolink nest survival (i.e., cutting hayfields after July 1) may create a situation where the nutritional quality of hay is too depleted for cattle. On the other hand, latitudinal gradients in optimal hay harvest timing in northern regions could functionally act like bird‐friendly mowing practices (i.e., delayed harvest), without necessitating human‐mediated intervention or, presumably, sacrificing nutritional quality of later‐cut forage. Outside of the 1990s, latitude may not have been identified as an important driver of Bobolink population trends if, for example, latitudinal difference in photoperiod or climate‐driven gradients in temperature were overridden by weather events that did not manifest on a north‐south gradient, such as precipitation.

Our derived indicator of hayfield composition was slightly negative over the entire study period, in contrast to results of some other studies (McCracken, 2005; Mussel, Schmidt, Ethier, & Doug, 2013). One reason for this discrepancy may be that we corrected our indicator for area. Previous studies that assessed hay harvest timing and frequency also failed to establish relationships between hayfield composition and Bobolink population trends (Corace et al., 2009; Herkert, 1997), suggesting that our result is not an artifact of how the predictor variable was derived.

Results presented here corroborate those of Hill et al. (2014) and Perlut (2014) in that overall pesticide use on the breeding grounds did not appear to be an important predictor of Bobolink population declines (but see Mineau & Whiteside, 2013). This could be due to, for examples, an overall decrease in the use of pesticides, or the general reduction in the toxicity of pesticides in the past several decades (Mineau & Whiteside, 2006). Future efforts to collect and model spatially and temporally explicit data on the amount, type, and application rates of various pesticides used per crop types, and their lethal, sublethal, and indirect effects on food resources and refuges, would greatly improve our understanding of pesticide risk to avian populations.

Our models primarily addressed how the loss and degradation of grassland habitat during the breeding season may affect Bobolink population trends, as these factors are within the management jurisdictions of local governments. However, Bobolinks live the majority of the year away from the breeding grounds, such that changes to migratory and wintering habitats may have important effects on population dynamics (Webster, Marra, Haig, Bensch, & Holmes, 2002). In systems with strong migratory connectivity, habitat degradation on the nonbreeding grounds should result in high spatial variation in the breeding season population trends. Connectivity is weak between breeding and nonbreeding areas for Bobolinks; Bobolinks from across the breeding range synchronously congregate on the nonbreeding ground over a relatively small geographic range (Renfrew et al., 2013). Thus, if the important drivers of Bobolink population change operate on nonbreeding areas, we might anticipate some degree of spatial trend homogenization (as occurred only during 2006–2011). Thus, the strong spatial variation in population trends we observed is inconsistent with the hypothesis that nonbreeding ground effects are driving Bobolink population trends in Ontario. Local conditions in the breeding range not accounted for among our predictor variables may more likely explain the observed spatial structure of trends (Bled et al., 2013).

We chose to use an irregular lattice for our CAR model because it enabled calculation of Bobolink population trends for agricultural census divisions, the spatial units at which land‐use statistics were available for testing our alternative hypotheses. However, different neighborhood structures are available, the selection of which can be critical for spatially explicit parameter estimation (Thogmartin et al., 2004). We assumed that the spatial random effect depended only on the adjacent neighborhood structure, and not on the distance between areas. Thus, the neighborhood structure used here may not fully reflect underlying environmental autocorrelations. Alternative spatial models include regular grids (Bled et al., 2013), geostatistical interpolation (Diggle, Tawn, & Moyeed, 1998), continuous surface models (Kelsall & Wakefield, 2002), or the replacement of spatial autocorrelation structure with appropriate environmental covariates at a scale relevant to underlying biological processes (Thogmartin et al., 2004). Further, for CAR models, the standard error of the spatial effect can have lower precision for cells with fewer neighbors (i.e., those at the periphery of our study area; Law & Haining, 2004). As a result, better‐connected census divisions are more likely to be significant due to narrower CRI. Using a logistic regression, we did a post hoc assessment to determine whether significant trend estimates were found more often than expected in better‐connected neighborhoods. We did not find evidence to support this assertion (*p* = .88, unpublished data), suggesting our results are not influenced by variation in the standard error of the spatial effect.

With a growing interest in developing regionally tailored management plans for priority grassland species (Cooper, 2007), consideration ought to be given to finer‐scale mechanisms to develop effective and efficient policy options with potential to arrest or reverse declines. Regional variability in Bobolink population trends, and the predictors identified to drive them, suggests that a one‐size‐fits‐all approach to grassland bird conservation may not adequately address threats to populations. Our results therefore speak to the need for active adaptive management to accommodate complex environmental systems that are not constant in space and time (Walters & Holling, 1990). This framework would allow resource managers to incorporate and address uncertainty regarding the factors that account for population declines, including nonlinearity and nonstationarity, while reducing these uncertainties by deliberate experimentation through policy implementation.

## Conflict of Interest

None declared.
