# Supplementary material for: Spatiotemporal variation in mechanisms driving regional‐scale population dynamics of a Threatened grassland bird
Source: Ecol Evol. 2017 Apr 27;7(12):4152–62. doi: 10.1002/ece3.3004 (PMC5478086; doi:10.1002/ece3.3004)
Supplement: Supplementary file 1 [file ECE3-7-4152-s001.docx]

**Appendix 1**: Alternative hypotheses about mechanisms driving variation in Bobolink population trends

The following variables were selected *a priori* to represent (in some instances, aggregate) mechanisms with potential to affect vital rates, based on evidence in the published literature and/or from stakeholder consultation (Ethier and Nudds, *in press*).

*Habitat amount*

Habitat loss may directly and indirectly influence reproductive success and mortality due to loss of nest sites or other resources. There is evidence for Bobolink consistent with these effects, albeit indirectly, in the form of temporal and spatial correlations between agricultural land-use data and breeding bird census data at various spatial scales (e.g., Murphy 2003; Corace et al. 2009). If Bobolink abundance trends in Ontario were driven by change in habitat amount, we anticipated positive correlations with percent change in hayfield and pasture amounts.

*Hayfield composition*

Once dominated by timothy (*Phleum pretense*) and clover (*Trifolium* spp.), hay is now often comprised of more alfalfa (*Medicago sativa;* Bollinger et al., 1990; Troy et al., 2005), with potentially two effects on Bobolink vital rates. First, alfalfa-dominated fields are cut earlier in the season (Barnes et al., 2007), putting Bobolink at greater risk of direct nest loss from mowing and raking, or loss of flightless young to subsequent predation on exposed nests (i.e., decline in habitat quality; Bollinger et al., 1990; Nocera et al., 2005; Perlut, 2007). Second, alfalfa-dominated hayfields are less attractive to Bobolinks than grass-dominated fields, effectively lessening the amount of available habitat (i.e., habitat loss; Bollinger, 1995). We therefore anticipated that, if Bobolink abundance trends were driven by changes in the ratio of grass: alfalfa (i.e., hayfield composition), we expect positive correlations with percent change in our hayfield indicator of hayfield composition.

*Cattle stocking density*

Cattle grazing can account for up to 100% of Bobolink nest failures due to trampling, bedding, and repeated nest disturbance (Kerns et al., 2010; MacDonald, 2014; Perlut et al., 2006; Perlut and Strong, 2011). Grazing may also indirectly degrade nesting habitat (Owens and Myres, 1973), increase nest predator abundance (Koper and Schmiegelow, 2007), and facilitate brood parasitism (Goguen and Mathews, 2001). In general, nest and habitat destruction tends to increase with grazing pressure (Jensen et al., 1969). We anticipated that, if Bobolink abundance trends were affected by any of these cattle induced mechanisms, then there will be a negative correlation with percent change in our indicator of cattle stocking density.

*Pesticide use*

Pesticides have been linked to population declines of grassland birds in the UK (Bright et al., 2008; Newton, 2004) and North America (Mineau et al., 2005). Pesticides can cause bird mortality as a result of direct exposure, secondary sub-lethal effects that alter behaviour or physiology, or indirect effects resulting from the elimination of food sources or refuges (Pimentel et al., 1992). While pesticide inputs into agricultural grassland are generally low relative to orchards, sod and vegetable crops, alfalfa carries the third highest lethal risk to birds of any crop based on pesticide use (Mineau and Whiteside 2006). In addition, only a small proportion of cropland needs to be treated with a dangerous pesticide to affect overall bird population trends, as residual pesticides and pesticide drift may extend the risk of pesticide use into adjacent fields (Mineau et al. 2005, Mineau and Whiteside 2013). Further, while a rise in seed treatments may not cause direct mortality of birds, they can have major indirect effects on prey resource abundance (reviewed by Bright et al. 2008). We anticipated that, if Bobolink abundance trends were influenced by pesticide use through any of the aforementioned mechanisms, then there will be a negative correlation with percent change in our indicator of pesticide use.

*Human population growth*

Variation in Bobolink abundances may result from human activities that directly affect mortality or displace birds. However, the relative effects of different factors, and the consequences for bird population dynamics, remain largely unquantified (Calvert et al., 2013). We modelled regional trends in human population size as an index of human activities that result in bird mortality from direct collisions with vehicles (Bishop and Brogan, 2013), secondary effect of roads on adjacent habitats (e.g., noise pollution; Reijnen & Foppen 2006), increased mortality from collisions with human structures (Machtans et al., 2013; Rioux et al., 2013; Zimmerling et al., 2013), and greater densities of cats and other synanthropic predators (Blancher 2013). We anticipated that, if Bobolink abundance trends were influenced by the aforementioned sources of mortality, then there will be a negative correlation with percent change in human population size.

*Habitat fragmentation*

Habitat fragmentation is implicated in population declines in some migrant birds (Askins et al., 1990; Faaborg et al., 1993) by causing distributional shifts away from and/or lower reproductive success near, habitat edges (Donovan et al., 1995). A number of factors may explain this response, including increased competition with edge-dominant species (Herkert 1991), increased rates of nest predation and parasitism (Johnson & Temple 1986), and aversion to habitat edges by birds that evolved in large contiguous tracts of habitat (Bollinger and Gavin, 2004; Temple and Cary, 1988). If Bobolink abundance trends were similarly negatively affected by edge effects, we anticipated a negative correlation with our static index of habitat fragmentation.

*Latitude*

Latitude, or variables correlated with latitude (e.g., growing-degree days), is a major determinant of rates of plant development, and thus of plant maturation (Fitter and Fitter, 2002)(Norris, 1986; Williams and Abberton, 2004). Thus, hayfields in southern Ontario are generally cut earlier than northern fields, increasing the likelihood of Bobolink nest failure (Nocera *pers. comm*.). Thus, we anticipated that Bobolink abundance trends would positively correlate with latitude (a static predictor).

**LITERATURE CITED**

Askins, R.A., Lynch, J.F., Greenberg, R., 1990. Population declines in migratory birds in the eastern North America. Curr. Ornithol. 7, 1–7.

Barnes, R.F., Nelson, C.J., Moore, K.J., Collins, M. (Eds.), 2007. Forages, The Science of Grassland Agriculture, 6 edition. ed. Wiley-Blackwell, Ames.

Bishop, C.A., Brogan, J.M., 2013. Estimates of avian mortality attributed to vehicle collisions in Canada. Avian Conserv. Ecol. 8. doi:10.5751/ACE-00604-080202

Blancher, P., 2013. Estimated Number of Birds Killed by House Cats (Felis catus) in Canada. Avian Conserv. Ecol. 8. doi:10.5751/ACE-00557-080203

Bollinger, E.K., 1995. Successional changes and habitat selection in hayfield bird communities. The Auk 112, 720–730.

Bollinger, E.K., Bollinger, P.B., Gavin, T.A., 1990. Effects of hay-cropping on eastern populations of the Bobolink. Wildl. Soc. Bull. 18, 142–150.

Bollinger, E.K., Gavin, T.A., 2004. Responses of nesting Bobolinks (Dolichonyx oryzivorus) to habitat edges. The Auk 121, 767–776.

Bright, J.A., Morris, A.J., Winspear, R., 2008. A review of indirect effects of pesticides on birds and mitigating land-management practices (No. RSPB Research Report No 28). Royal Society for the Protection of Birds, Sandy, Bedfordshire.

Calvert, A.M., Bishop, C.A., Elliot, R.D., Krebs, E.A., Kydd, T.M., Machtans, C.S., Robertson, G.J., 2013. A synthesis of human-related avian mortality in Canada. Avian Conserv. Ecol. 8, 11.

Corace, R.G., Flaspohler, D.J., Shartell, L.M., 2009. Geographical patterns in openland cover and hayfield mowing in the Upper Great Lakes region: implications for grassland bird conservation. Landsc. Ecol. 24, 309–323. doi:10.1007/s10980-008-9306-8

Donovan, T.M., Thompson, F.R., Faaborg, J., Probst, J.R., 1995. Reproductive Success of Migratory Birds in Habitat Sources and Sinks. Conserv. Biol. 9, 1380–1395. doi:10.1046/j.1523-1739.1995.09061380.x

Faaborg, J., Brittingham, M., Donovan, T., Blake, J., 1993. Habitat fragmentation in the temperate zone: a perspective for managers, in: Status and Management of Neotropical Migratory Birds, General Technical Report RM-229. Rocky Mountain Forest and Range Experiment Station, U.S. Department of Agriculture and Forest Services, Estes Park, Colorado, pp. 331–338.

Fitter, A.H., Fitter, R.S.R., 2002. Rapid Changes in Flowering Time in British Plants. Science 296, 1689–1691. doi:10.1126/science.1071617

Goguen, C.B., Mathews, N.E., 2001. Brown-Headed Cowbird Behavior and Movements in Relation to Livestock Grazing. Ecol. Appl. 11, 1533–1544. doi:10.2307/3060937

Herkert, J.R., 1991. An ecological study of the breeding birds of grassland habitats within Illinois [WWW Document]. URL http://hdl.handle.net/2142/23188

Jensen, H.P., Rollins, D., Gillen, R.L., 1969. Effects of cattle stock density on trampling loss of simulated ground nests. Wildl. Soc. Bull. 18, 71–74.

Johnson, R.G., Temple, S.A., 1986. Assessing habitat quality for birds nesting in fragmented tallgrass prairies, in: Wildlife 2000: Modeling Habitat Relationships of Terrestrial Vertebrates. University of Wisconsin Press, Madison, Wisconsin, pp. 245–249.

Kerns, C.K., Ryan, M.R., Murphy, R.K., Thompson, F.R., Rubin, C.S., 2010. Factors affecting songbird nest survival in northern mixed-grass prairie. J. Wildl. Manag. 74, 257–264. doi:10.2193/2008-249

Koper, N., Schmiegelow, F.K.A., 2007. Does Management for Duck Productivity Affect Songbird Nesting Success? J. Wildl. Manag. 71, 2249–2257. doi:10.2193/2006-354

MacDonald, N., 2014. The effects of rotational grazing and hay management on the reproductive success of Bobolink and Eastern Meadowlark in eastern Ontario (M.Sc. Thesis). Trent University, Peterborough, Ontario.

Machtans, C.S., Wedeles, C.H.R., Bayne, E.M., 2013. A first estimate for Canada of the number of birds killed by colliding with building windows. Avian Conserv. Ecol. 8. doi:10.5751/ACE-00568-080206

McGee, B., Berges, H., Beaton, D., 2010. Survey of pesticide use in Ontario, 2008: Estimates of pesticides used on field crops, fruit and vegetable crops, and other agricultural crops. Ministry of Agriculture, Food and Rural Affairs, Toronto, Ontario, Canada.

Mineau, P., Downes, C.M., Kirk, D.A., Bayne, E., Csizy, M., 2005. Patterns of bird species abundance in relation to granular insecticide use in the Canadian prairies. Ecoscience 12, 267–278. doi:10.2980/i1195-6860-12-2-267.1

Murphy, M.T., 2003. Avian population trends within the evolving agricultural landscape of eastern and central United States. The Auk 120, 20–34.

Newton, I., 2004. The recent declines of farmland bird populations in Britain: an appraisal of causal factors and conservation actions. Ibis 146, 579–600.

Nocera, J.J., Parsons, G.J., Milton, G.R., Fredeen, A.H., 2005. Compatibility of delayed cutting regime with bird breeding and hay nutritional quality. Agric. Ecosyst. Environ. 107, 245–253. doi:10.1016/j.agee.2004.11.001

Norris, I.B., 1986. Temperature response and flowering of white clover (Trifolium repens) varieties in controlled environments and the field. Ann. Appl. Biol. 108, 659–665. doi:10.1111/j.1744-7348.1986.tb02005.x

Owens, R.A., Myres, M.T., 1973. Effects of agriculture upon populations of native passerine birds of an Alberta fescue grassland. Can. J. Zool. 51, 697–713. doi:10.1139/z73-104

Perlut, N.G., 2007. Effects of hayfield management on grassland songbirds: behavioral responses and population processes. The University of Vermont.

Perlut, N.G., Strong, A.M., 2011. Grassland birds and rotational-grazing in the northeast: Breeding ecology, survival and management opportunities. J. Wildl. Manag. 75, 715–720. doi:10.1002/jwmg.81

Perlut, N.G., Strong, A.M., Donovan, T.M., Buckley, N.J., 2006. Grassland songbirds in a dynamic management landscape: behavioral responses and management strategies. Ecol. Appl. Publ. Ecol. Soc. Am. 16, 2235–2247.

Pimentel, D., Acquay, H., Biltonen, M., Rice, P., Silva, M., Nelson, J., Lipner, V., Giordano, S., Horowitz, A., D’Amore, M., 1992. Environmental and Economic Costs of Pesticide Use. BioScience 42, 750–760. doi:10.2307/1311994

Reijnen, R., Foppen, R., 2006. Impact of road traffic on breeding bird populations, in: Davenport, P.J., Davenport, J.L. (Eds.), The Ecology of Transportation: Managing Mobility for the Environment, Environmental Pollution. Springer Netherlands, pp. 255–274.

Rioux, S., Savard, J.-P.L., Gerick, A.A., 2013. Avian mortalities due to transmission line collisions: a review of current estimates and field methods with an emphasis on applications to the Canadian electric network. Avian Conserv. Ecol. 8. doi:10.5751/ACE-00614-080207

Temple, S.A., Cary, J.R., 1988. Modeling Dynamics of Habitat-Interior Bird Populations in Fragmented Landscapes. Conserv. Biol. 2, 340–347.

Troy, A.B., Strong, A.M., Bosworth, S.C., Donovan, T.M., Buckley, N.J., Wilson, M.L., 2005. Attitudes of Vermont dairy farmers regarding adoption of management practices for grassland songbirds. United States Geological Survey.

Williams, T.A., Abberton, M.T., 2004. Earlier flowering between 1962 and 2002 in agricultural varieties of white clover. Oecologia 138, 122–126. doi:10.1007/s00442-003-1407-0

Zimmerling, J.R., Pomeroy, A.C., d’Entremont, M.V., Francis, C.M., 2013. Canadian Estimate of Bird Mortality Due to Collisions and Direct Habitat Loss Associated with Wind Turbine Developments. Avian Conserv. Ecol. 8. doi:10.5751/ACE-00609-080210
